# The effects of anterior vacuum disc on surgical outcomes of degenerative versus spondylolytic spondylolisthesis: at a minimum two-year follow-up

**DOI:** 10.1186/1471-2474-15-329

**Published:** 2014-10-02

**Authors:** Tung-Yi Lin, Jen-Chung Liao, Tsung-Ting Tsai, Meng-Ling Lu, Chi-Chien Niu, Wen-Jer Chen, Lih-Hui Chen

**Affiliations:** Department of Orthopedic Surgery, Chang Gung Memorial Hospital, Chang Gung University, No._5, Fu-Shin Street, Kweishian, Taoyuan 333 Taiwan

**Keywords:** Disc vacuum phenomenon, Degenerative spondylolisthesis, Isthmic spondylolisthesis, Posterior instrumentation, Posterolateral fusion

## Abstract

**Background:**

The vacuum phenomenon within the intervertebral disc usually represents disc degeneration. There are no reports in the English literature that focus on the effect of an anterior vacuum disc on surgical outcome of same-segment spondylolisthesis.

**Methods:**

Patients with degenerative spondylolisthesis (DS) or isthmic spondylolisthesis (IS) who underwent a spinal surgery between January 2005 and December 2006 were reviewed. Patients who met certain criteria, including (1) only mono-segment spondylolisthesis, (2) gas air within the disc space of the spondylolisthesis segment on preoperative radiographs, (3) having received posterior decompression, posterior pedicle screw fixation, and posterolateral fusion, and (4) at least 12 months of follow-up radiographs available to define the posterolateral fusion rate, were enrolled into the study. Four radiographic parameters (disc height, translation, intradiscal angle, segmental angle) were assessed. Two-year postoperative radiographs were used to determine whether the posterolateral segment was fused or not. Clinical outcome and complications during the follow-up period were documented.

**Results:**

Incidence of the disc vacuum phenomenon was significantly higher in the IS group than in the DS group (p < 0.001). The IS group had more listhesis and a narrower disc height on preoperative static radiographs; however, the DS group had a more prominent angle and listhesis change in preoperative dynamic variables. The posterolateral fusion rate was significantly higher in the IS group (p = 0.019). The preoperative Oswestry Disability Index (ODI) score, the final ODI, and the ODI difference were similar between groups. More excellent and good results were seen in the IS group. Besides, better final ODI and results were seen in the bilateral fusion group than in the nonfusion group.

**Conclusion:**

The disc vacuum phenomenon is not equal to anterior instability absolutely. Determination of stability or instability in a vacuum disc should be considered by a combination of dynamic radiographs. In the present study, vacuum discs in the DS group showed more instability and a higher posterolateral pseudoarthrosis rate.

**Electronic supplementary material:**

The online version of this article (doi:10.1186/1471-2474-15-329) contains supplementary material, which is available to authorized users.

## Background

Disc structure has an important role in supporting the anterior part of a motion segment. The vacuum phenomenon refers to gas formation within the disc space. A vacuum disc is considered to be at an advanced stage of disc degeneration and a source of low back pain[1]. Kasai et al. studied the effect of changes in weather or barometric pressure on patients with a vacuum disc and concluded that low back pain is easily induced by a vacuum disc[2]. Because of the lack of material inside the disc, a vacuum disc is regarded as not contributing anterior support to the motion segment, and is treated as a sign of instability by some authors[3–5]. Furthermore, a prominent vacuum disc has demonstrated a close relationship with sagittal translation, which is an important sign of instability, that results in prominent low back symptoms[6, 7].

Spondylolisthesis is the anterior displacement of one vertebra over the caudal vertebra. There are five types of spondylolisthesis, based on the causes of slippage: congenital, dysplastic, isthmic, traumatic, and degenerative. The isthmic type and the degenerative type are the two most common forms of spondylolisthesis. Surgery is usually reserved for patients with low back pain and/or sciatica after failure of conservative treatment. With the development of lumbar pedicle screws in the 1990s, decompression with instrumented fusion has become a widely accepted surgical option for patients with spondylolithesis. The posterolateral fusion (PLF) rate has been reported to be 68% to 82% in instrumented patients, while overall clinical satisfactory results are 76% to 82%[8–11]. An anterior disc vacuum sign at the same level as spondylolisthesis is not rarely seen in practice. In theory, without performing interbody fusion, the residual disc would move persistently, reduce the PLF rate, and possibly engender a less satisfactory result. To our knowledge, however, there are no reports that focus on the effect of an anterior vacuum disc on surgical outcome of same-segment spondylolisthesis. Therefore, the purposes of the present study was to evaluate surgical outcome of patients with spondylolisthesis (degenerative or isthmic) combined with an anterior vacuum disc who underwent posterior decompression, posterior pedicle screw instrumentation, and PLF.

## Methods

After obtaining the approval from the Institutional Review Board of our institution, Chang Cung Medical Foundation, we retrospectively reviewed the records of patients with degenerative spondylolisthesis (DS) or isthmic spondylolisthesis (IS) who underwent a surgical procedure at our department between January 2008 and December 2010.

Patients enrolled into this study had to meet the following inclusion criteria: (1) only mono-segment spondylolisthesis, (2) gas air within the disc space of the spondylolisthesis segment on preoperative radiographs, (3) having received posterior decompression, posterior pedicle screw fixation, and PLF, and (4) at least 12 months of follow-up radiographs available to define the PLF rate. Patients who underwent multiple fusions, posterior interbody fusion, or anterior surgery, and those with an associated degenerative lumbar disease were excluded from this study. Each patient’s demographic and clinical data, including age, sex, surgical level, operation time, estimated blood loss, length of hospital stay, number of preoperative co-morbidities, and perioperative complications, were collected based on medical records. We also focused on any incidence of revision surgery related to the implant or adjacent segment degeneration.

### Study groups

**Figure 1:**
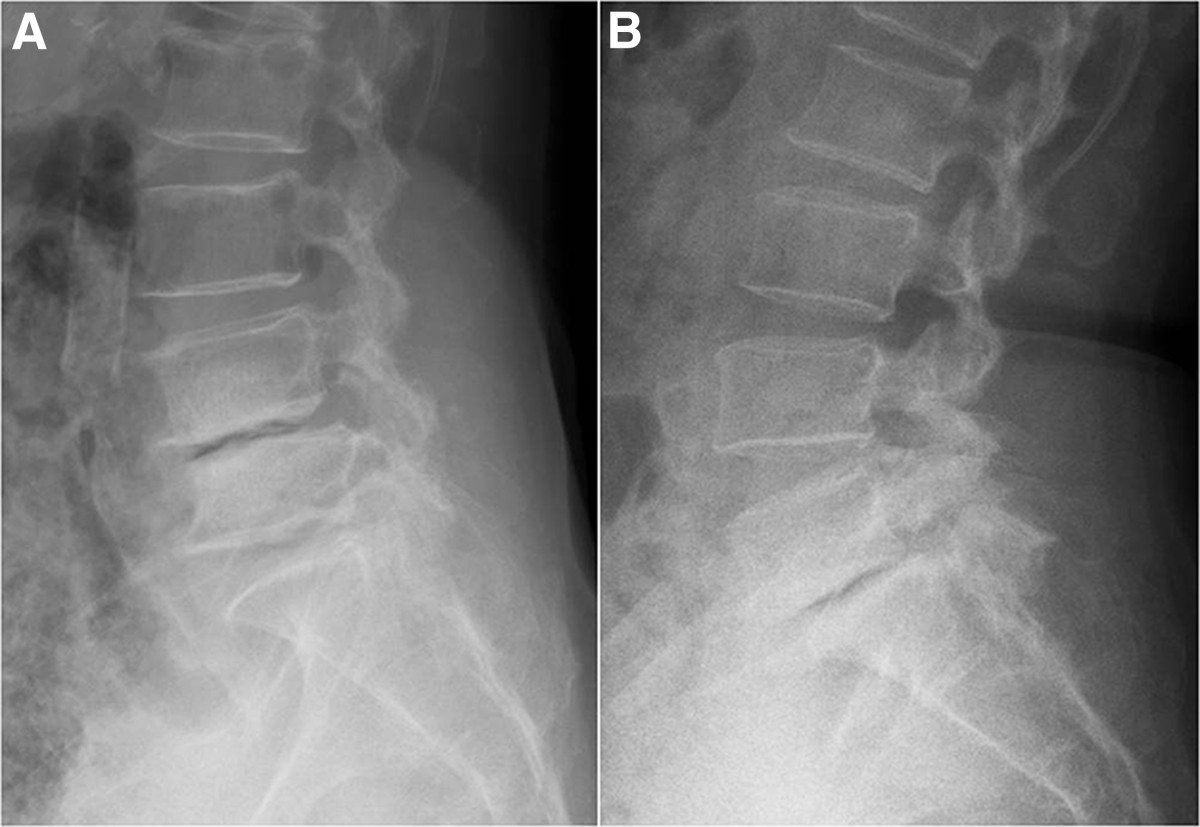
**Radiography of degenerative and spondylolytic spondylolisthesis.** **(A)** Radiograph, showing a case of degenerative spondylolisthesis (L4-5) with an anterior disc vacuum sign. **(B)** Radiograph, showing a case of isthmic spondylolisthesis (L5-S1) with an anterior vacuum phenomenon.

The enrolled patients were divided into two groups, based on the etiology of the spondylolisthesis: the IS group and the DS group (Figure 1).

### Evaluation

#### Radiographic parameters

**Figure 2:**
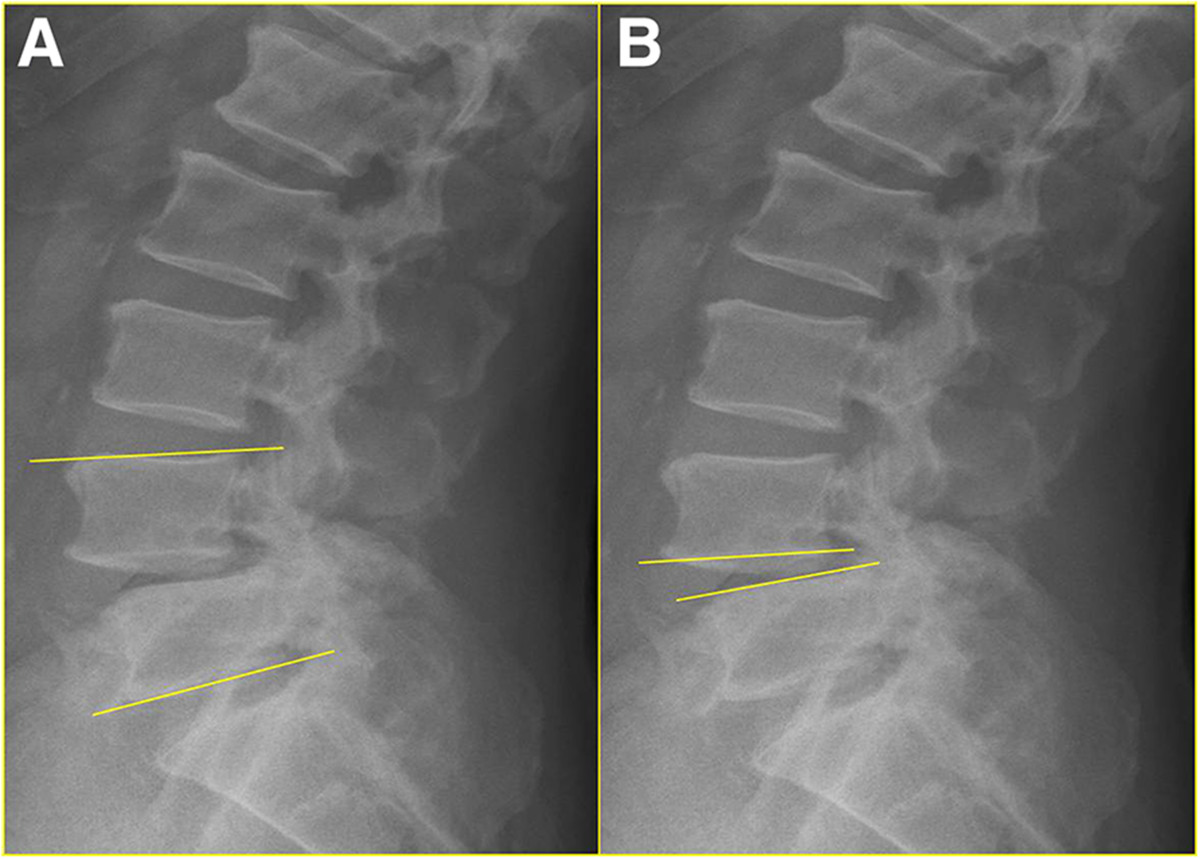
**Preoperative lateral radiographs were used to measure segmental lordosis (A) and intradiscal lordosis (B).**

**Figure 3:**
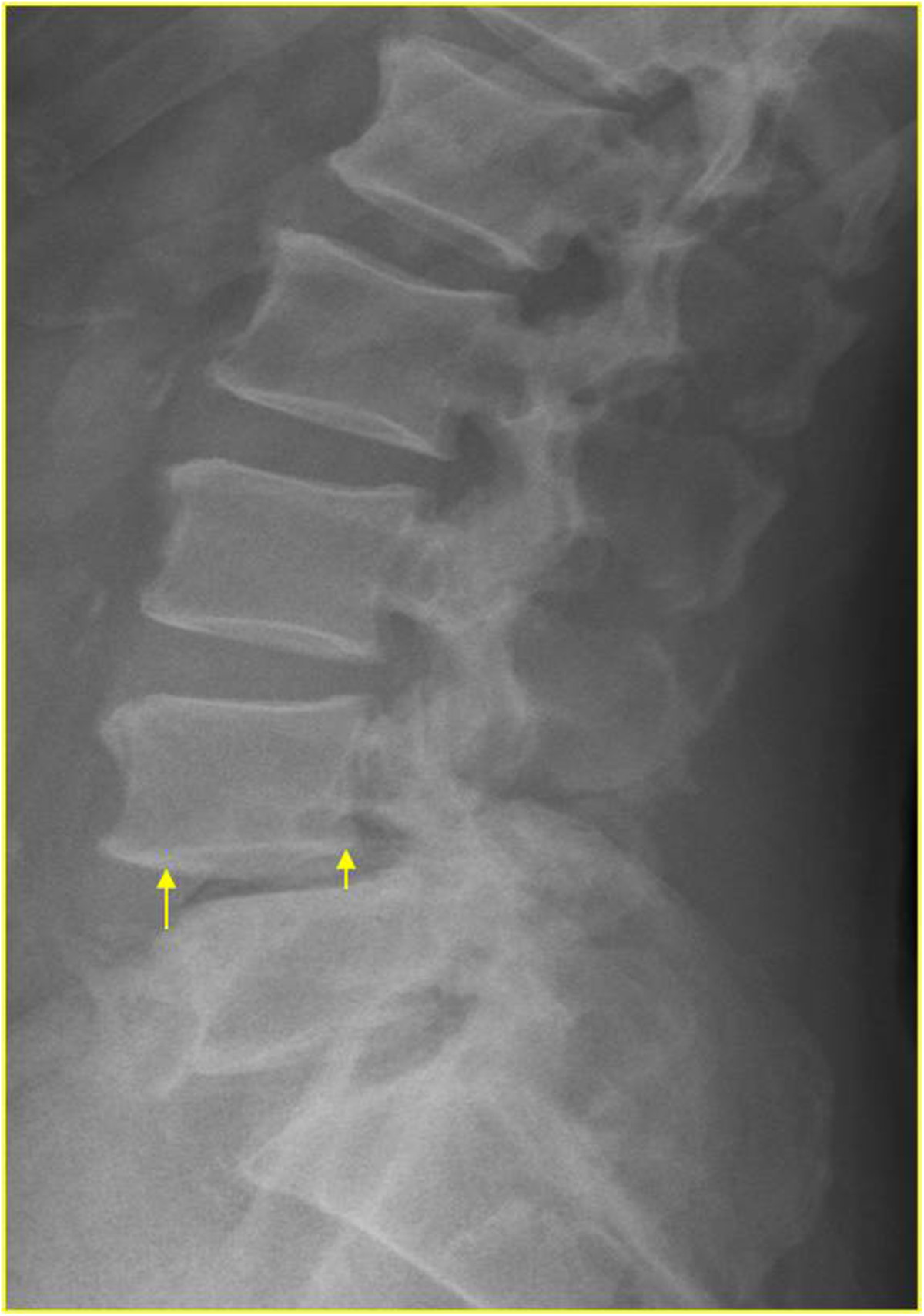
**Anterior and posterior disc heights were measured at the anterior and posterior aspects of the disc space, respectively.**

An independent reviewer provided a blind evaluation of the radiographs. Preoperative, postoperative, and final static standing lateral radiographs were measured. Radiographic parameters, including segmental lordosis, intradiscal lordosis, percentage of slipage, anterior disc height (ADH), and posterior disc height (PDH), were collected and compared between the two groups. Segmental lordosis was measured using a protractor, and defined as the angle between the cranial and caudal of endplates of the upper and lower vertebrae in the spondylolisthesis segment subjected to surgery (Figure 2). Intradiscal lordosis was defined as the angle between the caudal and cranial endplates of the upper and lower vertebrae (Figure 2). Percentage of slipage was measured using Tailard’s method[12]. ADH was measured as the distance between the most anterior point of the upper and lower end plates, while PDH was measured as the distance between the most posterior point of the upper and lower end plates (Figure 3). At the same time, we also measured preoperative flexion-extension standing lateral angle and slippage to evaluate mobility.

### Determination of fusion

**Figure 4:**
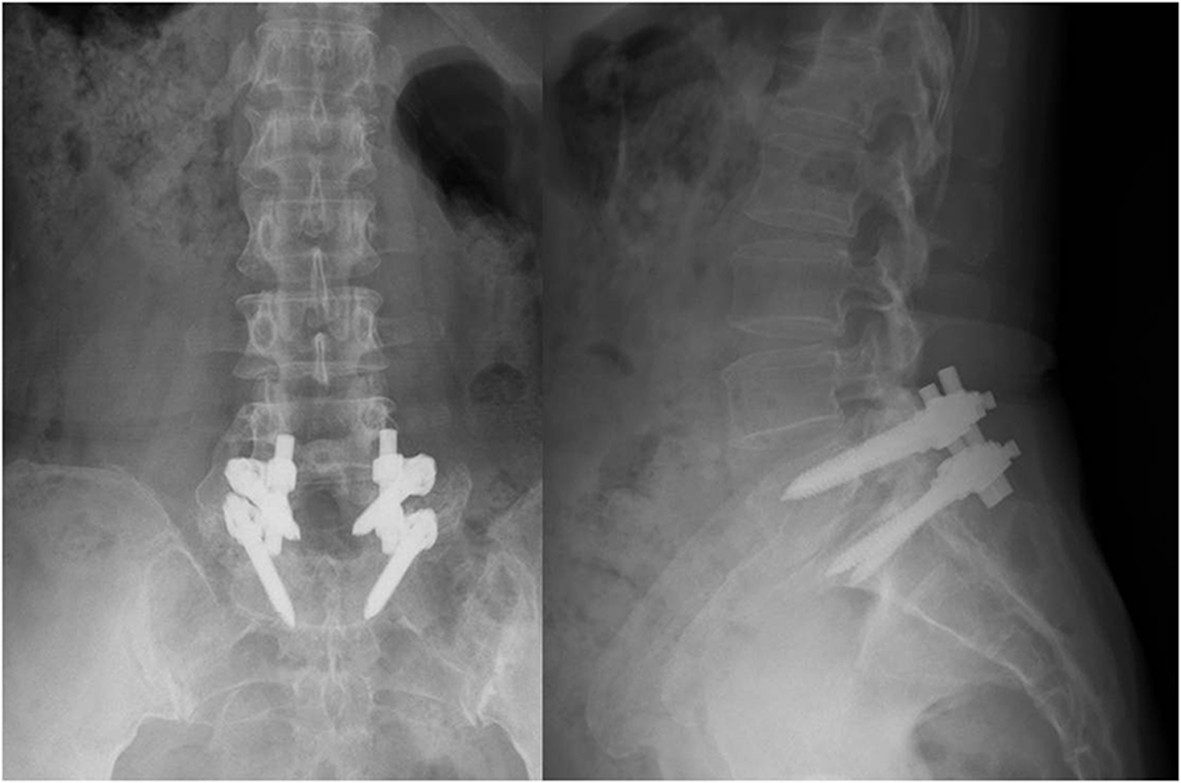
**Example of solid fusion after surgery.** A 55-year-old female underwent L5 laminectomy and L5-S1 instrumented posterolateral fusion. Two-year postoperative radiographs revealed fused L5-S1 segments and well-maintained implants.

We used standing anteroposterior view radiographs at the 2-year follow-up to determine inter-transverse fusion. Three independent observers who were not involved in the index operation performed this evaluation. Radiographic union was defined as having occurred when there was bridging bone formation between the inter-transverse processes without a gap (Figure 4). Successful inter-transverse fusion was determined when at least two of the three observers were in agreement.

### Clinical assessment

Objective clinial assessment was performed using the Oswestry Disability Index (ODI)[7] . In our department, all patients who underwent spinal surgery are asked to complete the pre-operative ODI questionnaire during their hospital admission before operation, while the final ODI questionnaire is completed in the outpatient clinic or by telephone interview. We obtained three types of ODI score: pre-operative ODI, final ODI, and ODI difference. ODI difference refers to the final ODI score subtracted from the pre-operative ODI score. The subjective clinical result was obtained using Brodsky’s criteria to evaluate the patient's self-reported satisfaction at the final follow-up, which assessed pain level, activity, and analgesic use[4]. Clinical results were categorized as excellent, good, fair, or poor. An excellent result indicated virtually no pain and increased daily activity level; a good result indicated improved symptoms, maintained daily activity level, and occasional pain that was relieved by short-term analgesics. A fair result indicated some improvement in symptoms, unchanged or decreased daily activity level, and low back pain or sciatica requiring frequent use of pain medication. A poor result indicated no change in or worsening of symptoms, decreased daily activity level, and daily analgesics use. Excellent and good results were defined as satisfactory, whereas fair and poor results were defined as unsatisfactory. All 42 patients including in this study provided their informed consent. Moreover, our research has adhered to the STROBE guidelines for observational studies.

## Results

Forty-two patients were included in this study: 22 in the DS group and 20 in the IS group. All patients underwent posterior decompression, one-segment posterior pedicle screw fixation, and one-segment PLF with iliac bone graft and laminectomy chip bone. The average age of the patients at eh time of surgery was higher in the DS group than in the IS group (65.6 vs. 57.3 years, p = 0.003). There were no statistically significant differences in gender distribution, operation time, blood loss, or hospital stay between the two groups. More patients in the DS group had comorbidity (68.1% vs. 40%), but the difference was not significant. The major surgical segment was L4-5 in the DS group and L5-S1 in the IS group. All patients in both groups had a vacuum signs on extension view radiographs. No patient in the DS group, but 8 in the IS group had a vacuum sign on flexion view. (p < 0.001). Three patients in the DS group developed perioperative complications: one suffered from postoperative sciatica because of pedicle screw malposition, and two had superficial wound infection. In the IS group, two patients had perioperative complications: one suffered from intraoperative incidental durotomy, while the other had superficial wound infection during admission. During the follow-up period, three patients in the DS group and one in the IS group developed adjacent segment disease and underwent revision surgery. There were no differences between the two groups in terms of incidence of perioperative complications, revision surgery, or adjacent segment disease (Table 1).

**Table 1:** **Patient demographics data**

	Degenerative group (n = 22)	Isthmic group (n = 20)	*p-* value
Age, mean ± SD, y	65.59 ± 7.74	57.30 ± 9.09	0.003
Sex, female/male, n	12/10	16/4	0.81
Operating time, mean ± SD, min	187.82 ± 37.49	191.65 ± 46.67	0.770
Blood loss, mean ± SD, cc	474.09 ± 331.31	387.25 ± 251.39	0.348
Hospital stay, mean ± SD, d	7.82 ± 2.40	8.10 ± 2.20	0.695
Level (n)			
L3-4	2	0	
L4-5	17	4	<0.001
L5-S1	3	16	
Vacuum type, n			
Static view	12	16	
Flexion view	0	8	<0.001
Extension view	22	20	
Comorbidity, n(%)	15(68.1)	8(40)	0.067
Perioperative complications, n(%)	3(13.6)	2(10)	0.716
Adjacent segment disease, n(%)	3(13.6)	1(5)	0.341
Revision surgery, n(%)	4(18.1)	2(10)	0.449
Fused segments, n(%)	25(56.8)	36(90)	0.019

### Radiographic results

In the DS group, 25 segments achieved successful PLF, for a fusion rate of 56.8% (25/44). In the IS group, the fusion rate was 90% (36/40), which was significantly higher than that of the DS group (p = 0.019).

In terms of preoperative static radiographic parameters, the DS group maintained a more lordotic intradisc angle, while the IS group had more listhesis (12.2 vs. 6.9 mm) and a narrower disc height. On flexion view, segmental and intradisc angles in the DS group turned to be less lordortic and slippage became more obvious; however, these parameters seemed not to change in the IS group. When the position changed to extension view, the DS group turned back to a more lordotic angle and had less listhesis, whereas the IS group seemed not to change again. We defined the differences between extension view and flexion view data as dynamic change. Dynamic variables such as slippage and lordotic change differed between the two groups: the DS group had a more prominent angle and listhesis change. The incidence of preoperative segmental and intradiscal kyphosis on flexion view were 5% and 25% in the IS group and 22.7% and 50% in the DS group, respectively; the differences were not significant. The incidence of lordotic change of more than 10° or slippage change of more than 4 mm was also higher in the DS group. Immediately after surgery, the DS group maintained a more lordotic intradisc angle (6.1° vs. 2.7°, p < 0.001), and had less slippage (5.7 vs. 8.73 mm, p = 0.002) and a greater disc height (ADH 8.8 vs. 4.6 mm p < 0.001; PDH 5. vs. 3.4 mm p < 0.001) compared with the IS group.

At the final follow-up, patients in both groups lost some degree of lordosis and disc height. The segmental angle in the IS group was more lordotic than that in the DS group. However, the intradisc angle was less lordotic, the disc height was narrower, and slippage remained more of a listhesis type in the IS group (Table 2).

**Table 2:** **Patient radiographic data**

	Degenerative group (n = 22)	Isthmic group (n = 20)	p ***-*** value
Preoperative static view			
Segmental angle, ^o^	10.70 ± 5.42	9.63 ± 5.70	0.538
Intradiscal angle, ^o^	4.74 ± 5.51	1.92 ± 3.01	0.049
Slippage, mm	6.85 ± 2.27	12.22 ± 5.15	< 0.001
ADH, mm	7.46 ± 3.09	4.12 ± 2.42	< 0.001
PDH, mm	5.16 ± 1.39	3.29 ± 1.85	0.001
Preoperative flexion view			
Segmental angle, ^o^	5.14 ± 6.72	10.53 ± 6.79	0.014
Intradiscal angle, ^o^	0.20 ± 6.38	1.09 ± 4.00	0.595
Slippage, mm	9.08 ± 2.26	10.84 ± 5.54	0.177
Preoperative extension view			
Segmental angle, ^o^	14.89 ± 6.95	14.25 ± 5.60	0.350
Intradiscal angle, ^o^	7.12 ± 5.152	2.65 ± 2.11	0.005
Slippage, mm	5.16 ± 2.72	10.11 ± 4.12	< 0.001
Postoperative static view			
Segmental angle, ^o^	12.61 ± 5.54	14.25 ± 5.60	0.350
Intradiscal angle, ^o^	6.11 ± 3.37	2.65 ± 2.11	< 0.001
Slippage, mm	5.65 ± 1.86	8.73 ± 3.79	0.002
ADH, mm	8.78 ± 2.29	4.62 ± 2.49	< 0.001
PDH, mm	5.48 ± 1.54	3.40 ± 1.63	< 0.001
Final static view			
Segmental angle, ^o^	10.65 ± 5.27	12.43 ± 5.21	0.279
Intradiscal angle, ^o^	9.14 ± 6.64	2.51 ± 2.23	0.095
Slippage, mm	6.97 ± 2.70	9.94 ± 4.15	0.008
ADH, mm	7.37 ± 2.75	4.25 ± 1.75	< 0.001
PDH, mm	4.62 ± 1.35	3.36 ± 1.60	0.009
Dynamic segmental angle change, ^o^	9.74 ± 4.74	1.65 ± 5.29	< 0.001
Dynamic intradiscal angle change, ^o^	6.92 ± 3.41	1.82 ± 4.46	< 0.001
Dynamic slippage change, mm	3.91 ± 1.53	0.73 ± 2.87	<0.001
Preoperative segmental kyphosis, n (%)	2 (9.1)	0 (0)	0.167
Preoperative intradiscal kyphosis, n (%)	3 (13.6)	2 (10)	0.716
Preoperative flexion segmental kyphosis, n (%)	5 (22.7)	1 (5)	0.101
Preoperative flexion intradiscal kyphosis, n (%)	11 (50)	5 (25)	0.096
Dynamic segmental lordotic change over 10°, n (%)	8 (36.6)	3 (15)	0.246
Dynamic intradiscal lordotic change over 10°, n (%)	3 (13.6)	0 (0)	0.230
Dynamic slippage change over 4 mm, n (%)	8 (36.4)	3 (15)	0.246

Data are presented as mean ± SD unless noted otherwise. Dynamic segmental angle change = preoperative extension segmental angle – preoperative flexion segmental angle; dynamic intradiscal angle change = preoperative extension intradiscal angle – preoperative flexion intradiscal angle; dynamic slippage change = preoperative flexion slippage – preoperative extension slippage. *Abbreviations: ADH* anterior disc height, *PDH* posterior disc height.

### Clinical results

At the latest interview, 13 patients in the DS group rated their results as excellent or good, 7 reported a similar status before and after surgery, and 2 rated their results as poor. In the IS group, 15 patients had excellent or good results, 4 had fair results, and 1 reported poor results. The success rate was higher in the IS group, but the difference was not significant (75.0% vs. 59.1%, p = 0.164).

The average preoperative ODI score was 51.45 ± 11.51 in the DS group, and 50.8 ± 9.62 in the IS group. The ODI scores of both groups decreased at the final follow-up by 24.27 ± 11.12 in the DS group, and by 25 ± 11.62 in the IS group; the difference was not significant (p = 0.841) (Table 3).

**Table 3:** **Clinical results (degenerative vs. isthmic)**

	Degenerative group (n = 22)	Isthmic group (n = 20)	p ***-*** value
Preoperative ODI score	51.45 ± 11.51	50.8 ± 9.62	0.847
Final ODI score	26.73 ± 9.51	25.3 ± 8.91	0.628
ODI score difference	24.27 ± 11.12	25 ± 11.62	0.841
Satisfaction, yes/no, n (%)	13/9 (59.09)	15/5 (75)	0.164

Data are presented as mean ± SD unless noted otherwise. Satisfaction was based on Brodsky’s criteria. *Abbreviation: ODI* Oswestry Disability Index.

In the DS group, 10 cases showed bilateral segment fusion, 5 showed unilateral segment fusion, and 7 cases showed nonfusion. In the IS group, 16 patients had bilateral segment fusion and 4 patients had unilateral segment fusion. The average preoperative ODI score was 51.77 ± 12.49 in the bilateral fusion group, 49.33 ± 7.12 in the unilateral fusion group, and 51.14 ± 5.54 in the nonfusion group. Final ODI scores between fusion and nonfusion groups were significant different (P =0.004). The success rate was higher in the fusion group, compared to the nonfusion group. (84.6% vs. 28.6% p = 0.002) (Table 4).

**Table 4:** **Clinical results (bilateral vs. unilateral fusion, bilateral vs. no fusion)**

	Bilateral (n = 26)	Unilateral (n = 9)	No fusion (n = 7)	Bilateral vs. unilateral p ***-*** value	Bilateral vs. no fusion p ***-*** value
Preoperative ODI score	51.77 ± 12.49	49.33 ± 7.12	51.14 ± 5.54	0.901	0.594
Final ODI score	22.62 ± 9.46	29.56 ± 5.23	34.29 ± 4.33	0.050	0.004
Satisfaction, yes/no, n (%)	22/4 (84.6)	4/5 (44.4)	2/5 (28.6)	0.058	0.002

Data are presented as mean ± SD unless noted otherwise. Satisfaction was based on Brodsky’s criteria. *Abbreviation: ODI* Oswestry Disability Index.

## Discussion

The pathogenesis of DS and IS are different. The primary lesion responsible for IS is a defect of the pars interarticularis. The proposed etiologies for this pars defect include chronic stress fracture, acute pars fracture, and chronic hyperextension stress on the elongated pars in childhood or adolescence. Because of the pars defect, the cephalic vertebra tends to move anteriorly, and the disc acts as a stabilizer to oppose the anteriorly directed shear force at the segment of spondylolisthesis[13, 14]. In humans, the disc begins to degenerate during the third decade of life, and adult slippage progression in spondylolisthesis is likely to develop during the fourth and fifth decades of life[15, 16]. In this study, all patients’ discs showed the vacuum phenomenon, representing advanced disc degeneration, which is why the average age at surgery in the IS group was 57 years (the sixth decade of life). In contrast, DS is different from that of IS. DS is believed to be initially triggered by posterior ligament and facet laxity, which promotes hypermobility. Thereafter, a decrease in proteoglycan and water content within the degenerated disc further alters the kinematics of the motion segment[17, 18]. To re-stabilize the motion segment, facet joint hypertrophy occurs, but might result in spinal canal stenosis.

Currently, PLF with instrumentation has gained popularity as a surgical treatment for DS. In general, short-term results are believed to be similar between noninstrumentation and instrumentation, but a higher PLF rate and better long-term results are seen with instrumentation[19]. In the current study, all 22 patients in the DS group underwent posterior decompression, instrumentation, and PLF, but the percentage of fused segments at 2-years postoperative was only 57%, which is far lower than that reported in previous studies[20–22]. These 22 patients all had a vacuum appearance on extension view, but without an air sign on flexion view, indicating the amount of air in the disc was not so abundant, and needed to be squeezed to appear during flexion-extension motion. Furthermore, the average segmental angle and listhesis change between flexion and extension views were 9.7° and 3.9 mm, respectively; we believe these 22 vacuum discs in the spondylolisthesis segment were in an unstable condition. Without anterior support, the pedicle screws would be subjected to excess stress, which would have contributed to more pseudoarthrosis and implant failure. Oda et al. performed a calf spine cadaver study using pedicle screws in five groups: intact + pedicle screws (I-PS), medial fascectomy + pedicle screws (MF-PS), total fascectomy + pedicle screws (TF-PS), partial discectomy + pedicle screws (D-PS), and partial discectomy + pedicle screws + interbody cage (D-PS-PLIF). The D-PS group had the highest strain between the screw and rod; in contrast, the D-PS-PLIF group demonstrated the lowest load at the screw-rod interface. The authors concluded that posterior pedicle screw fixation alone is adequate when anterior load sharing is preserved, but high pedicle screw strain and insufficient stability are encountered when anterior column support is damaged, which can be resolved by addition of an interbody cages[23].

On the other hand, the characteristics of the IS group were different from those of the DS group. All 20 patients in the IS group had a vacuum sign on extension view, 16 had a vacuum sign on static lateral view, and 8 had a vacuum sign on flexion view. Although the incidence of a vacuum disc on flexion view was higher (p < 0.001), and preoperative listhesis distance was longer in the IS group (12.0 vs. 6.9 mm, p < 0.001), the percentage of final fused segments was higher in the IS group (90% vs. 56.8%, p = 0.019). Disc degeneration with disc height narrowing is considered related to spinal instability[24]. The radiographic results showed a narrower disc height in the IS group on preoperative static view, postoperative static view and final static view (Table 2). However, in our series, narrower disc height in the IS group did not lead to a poor fusion rate or unsatisfactory clinical results. Many factors could explain this result. First, based on studies of the natural history of the disc and spondylolisthesis, Matsunaga et al. demonstrated that when disc height is collapsed, there is a natural tendency to restabilize the motion segment and as a result, spondylolisthesis becomes less likely to progress[25]. Murata et al. performed a study of 109 patients with low back pain and/or sciatica by analyzing lumbar disc height, horizontal displacement, and angular displacement on plain radiographs, and comparing these findings with disc degeneration on MRI[5]. They found that severe disc degeneration was less significantly related to angular displacement, and had a tendency to stabilize the motion segment. We believe the degenerative discs in the spondylolisthesis segment in the IS group had entered the third phase (stabilization) of the Kirkaldy-Willis degeneration cascade[26], thereby contributing to motion segment stabilization and enhancing the surgical fusion rate. Second, from the perspective of dynamic change of the motion segment, the dynamic motion of spondylolisthesis in the IS group was more stable compared with that in the DS group. In the IS group, dynamic change of the segmental angle was approximately 1.7°, whereas this dynamic change was 9.7° in the DS group (p < 0.001). This phenomenon was also seen in intradisc angle change (1.8° vs. 6.9°, p < 0.001). In addition, dynamic slippage change was longer in the DS group (3.9 vs. 0.7 mm, p < 0.001). These findings reveal that patients in the DS group sustained more anterior instability. Indeed, we also found that some patients in the IS group (data not shown in the present study) had paradoxical motion, as Oh et al. described[27]. In paradoxical movement, flexion reduces spondylolisthesis while extension increases anterior listhesis. This special movement in spondylolytic spondylolisthesis is usually explained by the “impingement” theory[28]. The average slippage change in dynamic motion was limited (only 0.7 mm) in the IS group, which could be explained by greater stability of the anterior segment, and by the paradoxical motion in the IS group. Even if we used dynamic angle change >10° and dynamic anterior listhesis change >4 mm as a sign of instability, the incidence was higher in the DS group, though not significantly.

Farfan et al. emphasized that a disc below the pars defect easily becomes degenerated due to rotatory and anterior shearing force on the disc[29]. Virta and Ronnemaa found that disc height at the slippage level was inverted to the degree of IS in middle-aged patients[30]. Floman observed 18 adult patients with lumbosacral IS and found that slippage progression started from the third decade of life, with progression from 9% to 30% during a period of 2 to 20 years[15]. When these patients became symptomatic and candidates for surgery, marked disc degeneration, including narrowing of the disc space, osteophyte formation, and vacuum sign, was noted to be coincident at the listhesis level. Based on the literature review above and our present study, we believe the IS disc becomes degenerative rapidly after the third or fourth decade of life. By the time these patients are symptomatic and require surgical intervention, these vacuum discs with low intervertebral height have passed the unstable stage, so posterior instrumentation and PLF are adequate for them. In conclusion, the disc vacuum phenomenon indicates there is gas formation inside the intervertebral space. This condition reveals disc degeneration, but is not absolutely equal to anterior instability. A degenerative disc of this type might remain at the unstable stage or enter the re-stabilization phase, which can be determined by dynamic (flexion-extension) radiographs.

Comparison between DS and IS is debatable because of their different pathogenesis, which may lead to some bias. However, both groups display spondylolisthesis on radiography, and can have an anterior vacuum disc at the same-segment of spondylolisthesis. The purposed of this study was to present the results of patients with DS or IS combined with an anterior vacuum disc who underwent the same treatment, instrumented posterolateral fusion.

## Conclusions

In the present study, the behavior of the vacuum disc in the DS group showed more anterior instability than that in the IS group, which resulted in a higher posterolateral pseudoarthsosis rate and less satisfactory clinical result. Therefore, simultaneous interbody fusion with a cage might be a solution to overcome the anterior instability found in patients with DS and a vacuum disc, but this requires further study for confirmation.

## Authors’ original submitted files for images

Below are the links to the authors’ original submitted files for images. Authors’ original file for figure 1 Authors’ original file for figure 2 Authors’ original file for figure 3 Authors’ original file for figure 4

## Abbreviations

DS: Degenerative spondylolisthesis
IS: Isthmic spondylolisthesis
PLF: Posterolateral fusion
ADH: Anterior disc height
PDH: Posterior disc height
ODI: Oswestry Disability Index.
