# Supplementary figures and images for: The effects of anterior vacuum disc on surgical outcomes of degenerative versus spondylolytic spondylolisthesis: at a minimum two-year follow-up
Source: BMC Musculoskelet Disord. 2014 Oct 2;15:329. doi: 10.1186/1471-2474-15-329 (PMC4201703; doi:10.1186/1471-2474-15-329)

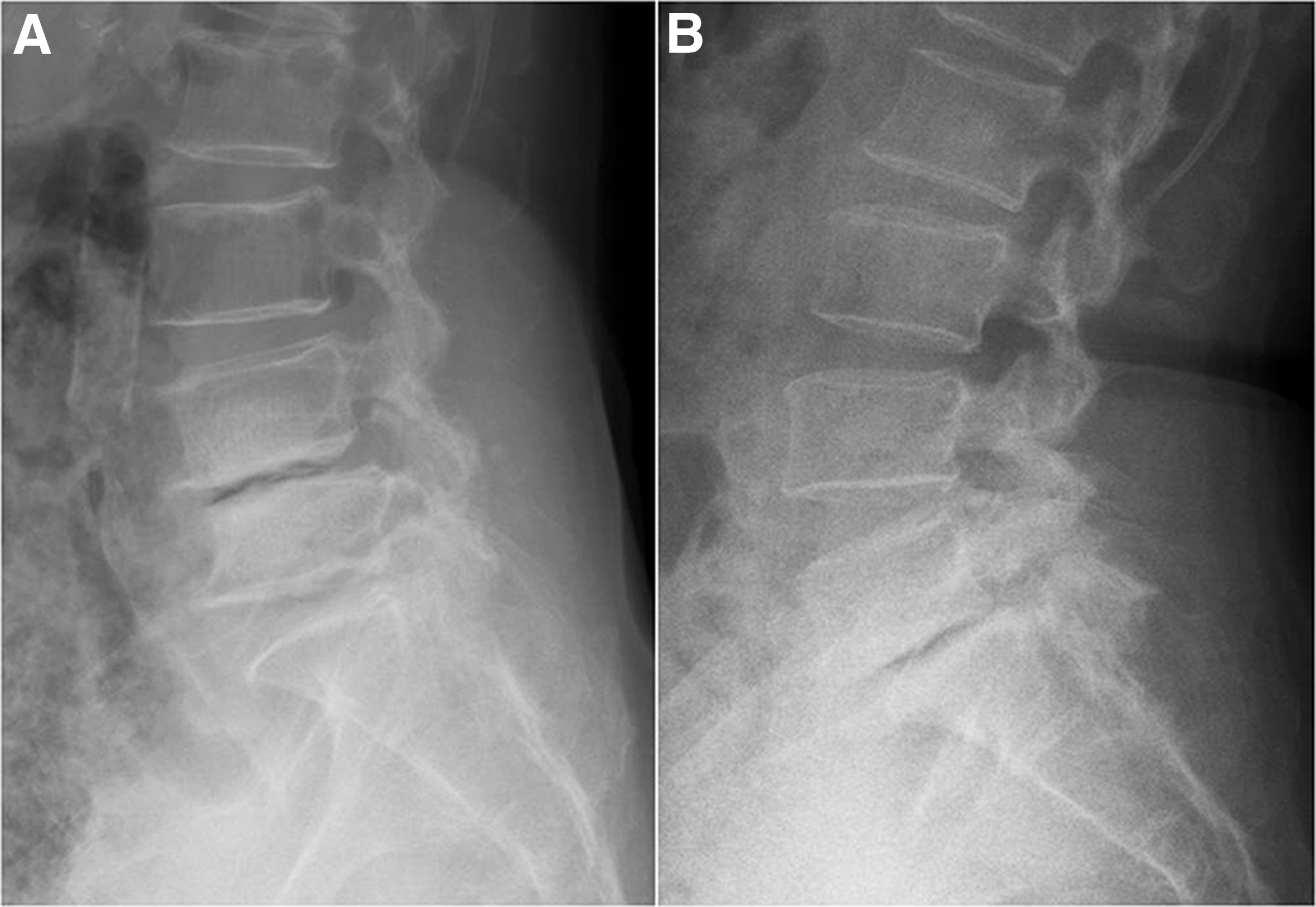

Supplement: Supplementary file 1 — Authors’ original file for figure 1 [file 12891_2013_2279_MOESM1_ESM.tif]

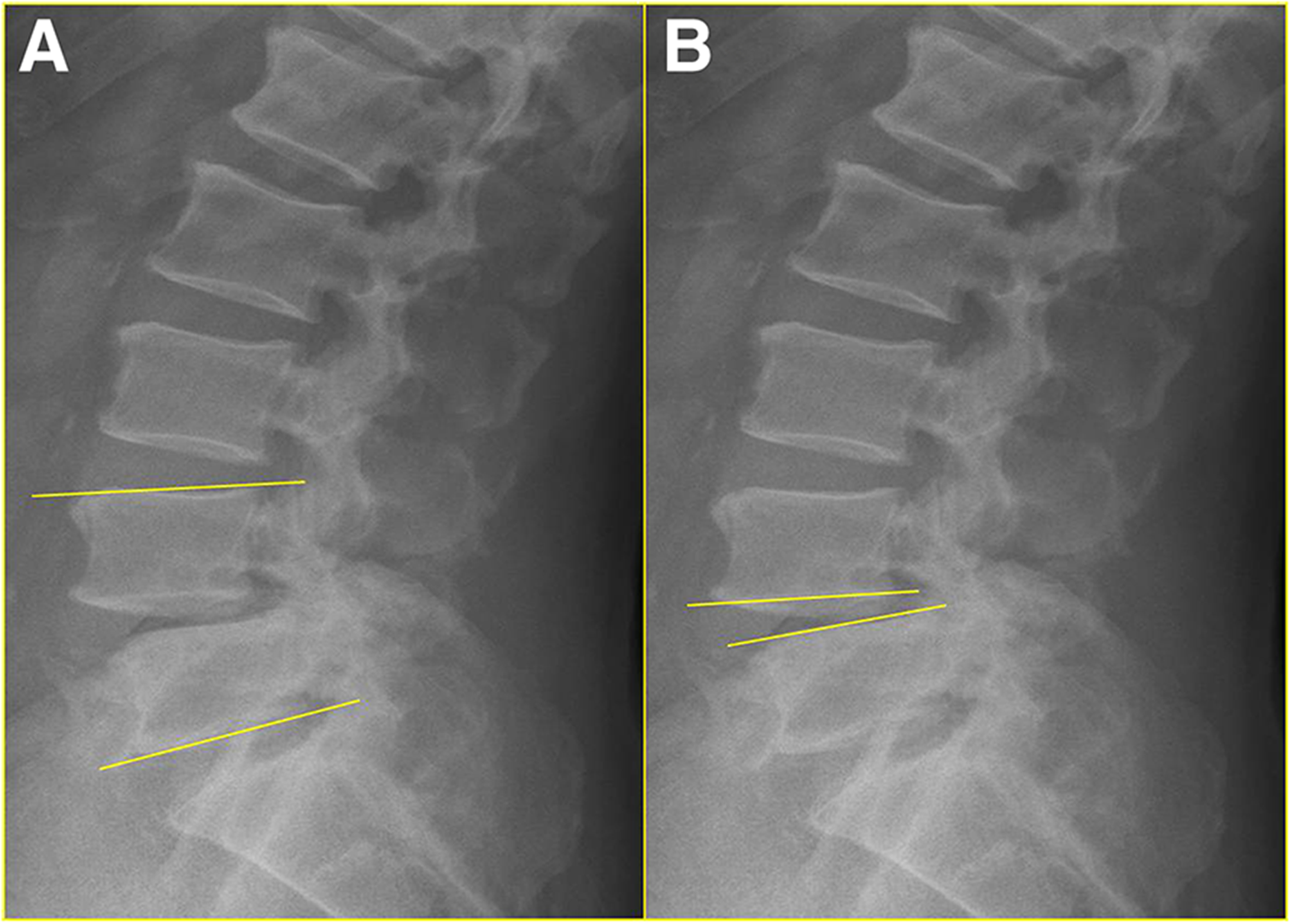

Supplement: Supplementary file 2 — Authors’ original file for figure 2 [file 12891_2013_2279_MOESM2_ESM.tif]

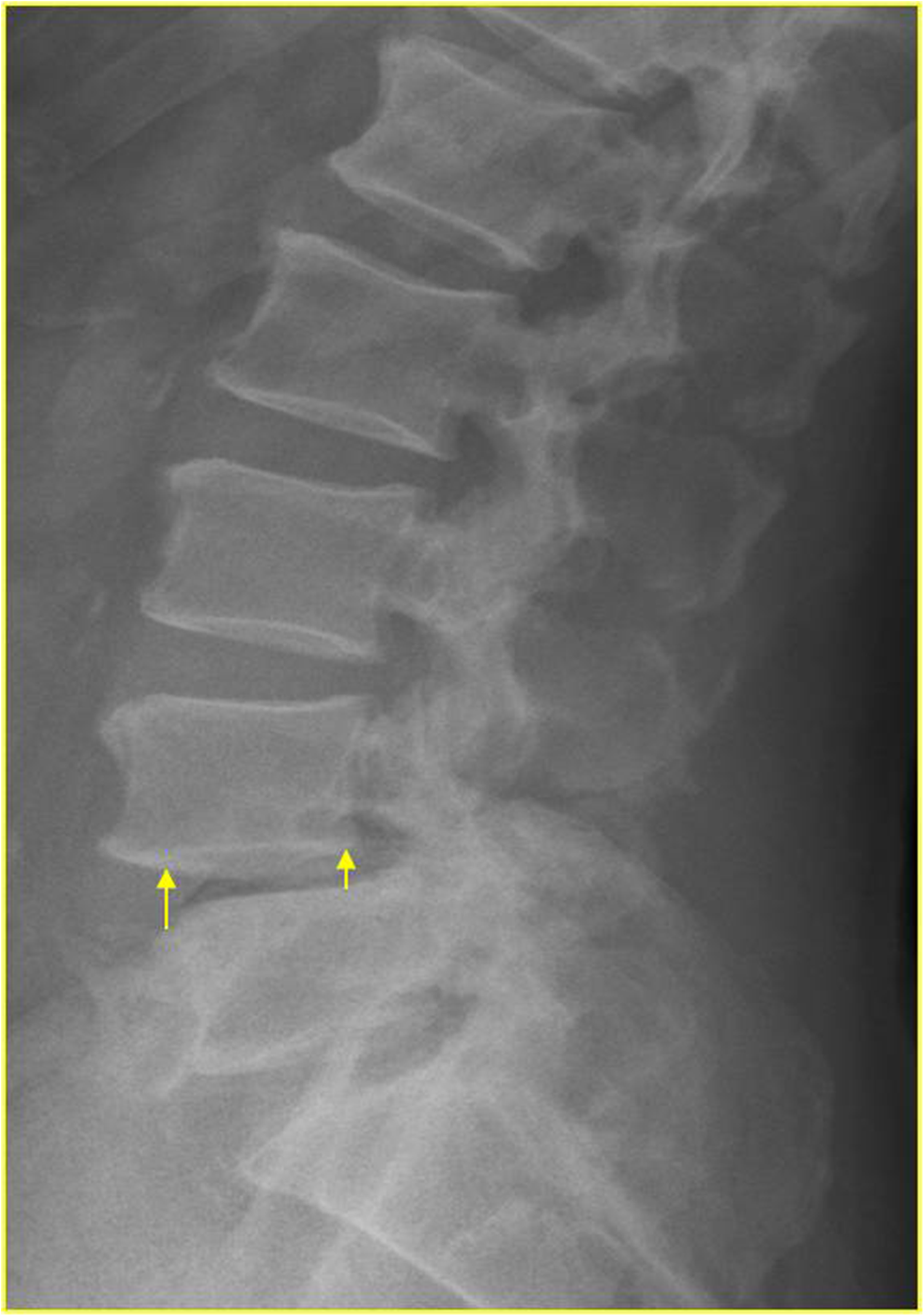

Supplement: Supplementary file 3 — Authors’ original file for figure 3 [file 12891_2013_2279_MOESM3_ESM.tif]

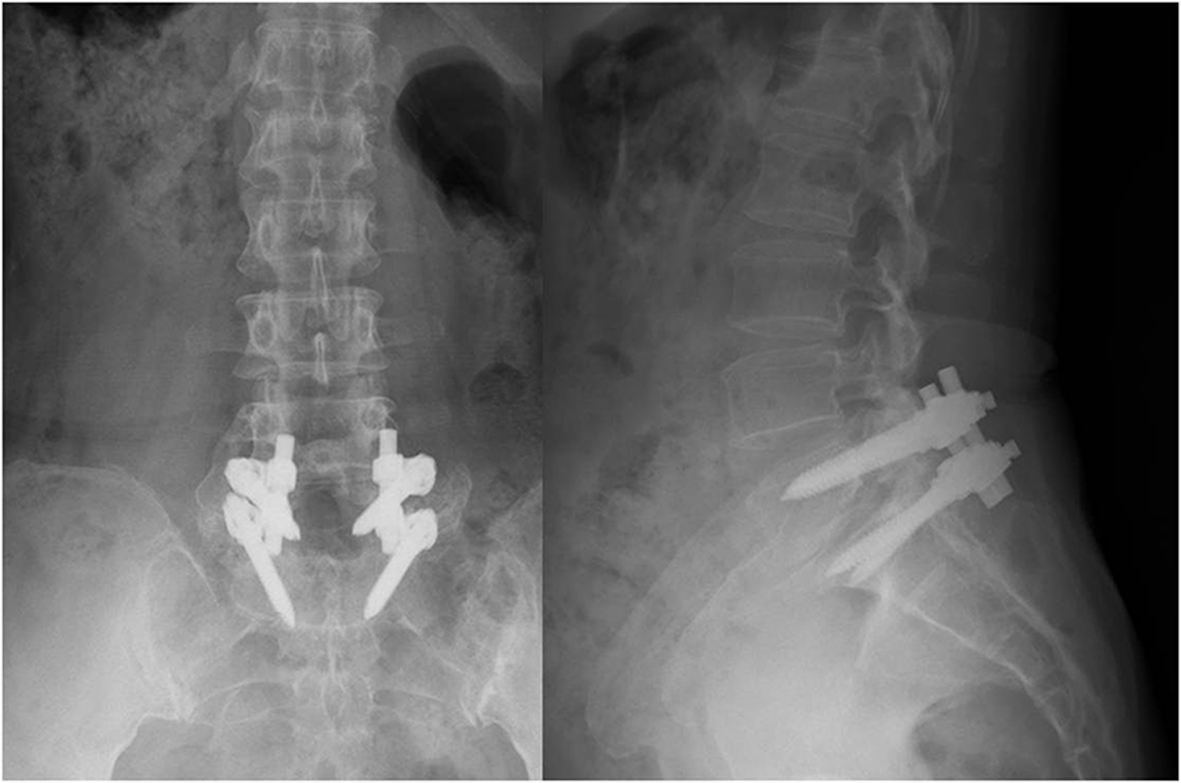

Supplement: Supplementary file 4 — Authors’ original file for figure 4 [file 12891_2013_2279_MOESM4_ESM.tif]
